# Geographic and Population Distributions of Human Immunodeficiency Virus (HIV)–1 and HIV-2 Circulating Subtypes: A Systematic Literature Review and Meta-analysis (2010–2021)

**DOI:** 10.1093/infdis/jiad327

**Published:** 2023-08-18

**Authors:** Alexandria Williams, Sonia Menon, Madeleine Crowe, Neha Agarwal, Jorne Biccler, Nicholas Bbosa, Deogratius Ssemwanga, Ferdinard Adungo, Christiane Moecklinghoff, Malcolm Macartney, Valerie Oriol-Mathieu

**Affiliations:** P95 Pharmacovigilance and Epidemiological Services, Leuven, Belgium; P95 Pharmacovigilance and Epidemiological Services, Leuven, Belgium; P95 Pharmacovigilance and Epidemiological Services, Leuven, Belgium; P95 Pharmacovigilance and Epidemiological Services, Leuven, Belgium; P95 Pharmacovigilance and Epidemiological Services, Leuven, Belgium; Medical Research Council/Uganda Virus Research Institute and London School of Hygiene and Tropical Medicine Uganda Research Unit, Entebbe; Medical Research Council/Uganda Virus Research Institute and London School of Hygiene and Tropical Medicine Uganda Research Unit, Entebbe; Kenya Medical Research Institute, Nairobi; Janssen-Cilag GmbH, Neuss, Germany; Janssen-Cilag Limited, High Wycombe, United Kingdom; Janssen Vaccines and Prevention, B.V., Leiden, The Netherlands

**Keywords:** HIV subtypes, HIV-1, HIV-2, epidemiology, prevalence

## Abstract

**Background:**

HIV poses significant challenges for vaccine development due to its high genetic mutation and recombination rates. Understanding the distribution of HIV subtypes (clades) across regions and populations is crucial. In this study, a systematic review of the past decade was conducted to characterize HIV-1/HIV-2 subtypes.

**Methods:**

A comprehensive search was performed in PubMed, EMBASE, and CABI Global Health, yielding 454 studies from 91 countries.

**Results:**

Globally, circulating recombinant forms (CRFs)/unique recombinant forms (URFs) accounted for 29% of HIV-1 strains, followed by subtype C (23%) and subtype A (17%). Among studies reporting subtype breakdowns in key populations, 62% of HIV infections among men who have sex with men (MSM) and 38% among people who inject drugs (PWIDs) were CRF/URFs. Latin America and the Caribbean exhibited a 25% increase in other CRFs (excluding CRF01_AE or CRF02_AG) prevalence between 2010–2015 and 2016–2021.

**Conclusions:**

This review underscores the global distribution of HIV subtypes, with an increasing prevalence of CRFs and a lower prevalence of subtype C. Data on HIV-2 were limited. Understanding subtype diversity is crucial for vaccine development, which need to elicit immune responses capable of targeting various subtypes. Further research is needed to enhance our knowledge and address the challenges posed by HIV subtype diversity.

## Teaser

Globally, circulating recombinant forms (CRFs) and unique recombinant forms (URFs) make up 29% of all HIV subtypes and have increased by around 8% from 2010 to 2021. Among key populations, CRF/URFs account for the largest proportion of HIV infections.

Globally, 38.4 million people lived with human immunodeficiency virus (HIV) in 2021 [1]. There are 2 lineages of HIV—namely, HIV-1 and HIV-2—which originated from zoonotic transmission of simian immunodeficiency virus from nonhuman primates to humans [2, 3]. HIV-1 accounts for 95% of infections globally and is divided into 4 groups (M, N, O, and P), with group M containing 9 distinct subtypes, or clades [4, 5], the main ones being C, B, and A, which make up around 70% of the global distribution of HIV-1 [6]. Compared to HIV-1, HIV-2 is uncommon, slower progressing, and mostly concentrated in West Africa [7]. In 2021, 1.5 million new HIV infections and 650 000 AIDS-related deaths were reported [1]. Despite a reduction in incidence over the last few decades due to improved treatment and prevention efforts, this decline in infections has slowed over the past 5 years—especially in key populations: sex workers and their clients, men who have sex with men (MSM), people who inject drugs (PWIDs), transgender individuals, and prison inmates [8]. Understanding the prevalent HIV subtypes in these key populations is crucial for designing targeted prevention and intervention strategies. Overall, the diversity of HIV subtypes has important implications for vaccine development, where vaccines must elicit immune responses capable of effectively targeting the various subtypes of the virus.

The global geographical subtype distribution of HIV-1 is evolving over time and there has been a notable increase in newly emerging recombinants [6, 9]. Further recombination between subtypes results in circulating recombinant forms (CRFs) and unique recombinant forms (URFs), where CRFs are classified when found in at least 3 epidemiologically unlinked individuals, and URFs show no onward transmission outside a single individual [10]. Currently, there are 118 HIV-1 CRFs documented in the Los Alamos HIV database [11], with the 2 main ones—CRF01_AE and CRF02_AG—being most prevalent in Asia and Western Africa, respectively [4].

HIV-1 subtype surveillance is epidemiologically and clinically relevant, as these subtypes may be associated with different pathogenesis and resistance mechanisms, and has implications for vaccine development [12, 13]. One of the major barriers to developing a safe and effective vaccine is the high mutation and recombination rates that HIV undergoes during replication [14]. Furthermore, the geographic distribution of these clades poses challenges for vaccine design and implementation strategies, and whether it is better to prioritize a clade-matched or region-specific vaccine strategy [15]. For example, the mosaic vaccine uses bioinformatically optimized bivalent global mosaic antigens to expand immune coverage across diverse HIV-1 strains [16–18]. A phase 2b study of the mosaic vaccine (IMBOKODO; NCT03060629) did not show statistically significant protection against HIV-1 infection in young women at high risk living in sub-Saharan Africa. Similarly, another mosaic vaccine trial within MSM and transgender individuals in the Americas and Europe was discontinued at phase 3 due to a lack of efficacy in preventing HIV (MOSAICO; NCT03964415).

Due to the heterogenous and evolving nature of HIV, treatment, prophylactic measures, and vaccine development require up-to-date knowledge of HIV subtype distribution within populations at both the regional and global level. Previously, Hemelaar et al conducted a systematic literature review (SLR) to estimate geographic distributions of HIV-1 subtypes between 1990 and 2015, but did not investigate HIV-1 subtypes across key populations nor proportions of HIV-2 [6]. Another review by Bbosa et al summarized HIV-1 and HIV-2 global distributions between 1987 and 2018 [4]. Thus, this comprehensive review builds on their work by performing an updated SLR using multiple primary literature databases to provide a current snapshot of prevalent HIV-1 and HIV-2 clades across geographic regions and key populations.

## METHODS

This review was reported in accordance with the Preferred Reporting Items for Systematic Reviews and Meta-Analyses (PRISMA) guidelines [19]; a PRISMA diagram and checklist can be found in Supplementary Appendix 1. The protocol can be found online in the International Prospective Register of Systematic Reviews (PROSPERO) ID CRD42021258880 [20]; of note, the risk of bias (RoB) tool reported in the protocol was changed after submission to PROSPERO, which could not be further amended.

### Search Strategy and Selection Criteria

A systematic search of PubMed, Embase, and CABI Global Health was conducted for peer-reviewed publications reporting HIV-1 or HIV-2 subtype data between January 2010 and June 2021. The search query terms are specified in Supplementary Table 1, while the full query and methods are described in Supplementary Appendix I. In brief, we included publications in any language across all regions and populations, irrespective of age, sex, ethnicity, CD4 cell count, viral load, antiretroviral treatment, or coinfections. Papers were excluded if data were collected before 2010 or did not cover the full year of 2010, no baseline subtyping data were provided, paper type was a conference abstract, editorial/opinion, literature review, or case report/case series, or study design was an animal/postmortem or Bayesian modeling study. No gray literature (unpublished) was included.

### Screening and Data Extraction

A total of 1681 references were included for full-text screening, which was done by 2 reviewers, and a third reviewer resolved conflicts. Selected studies were extracted using DistillerSR [21] by 1 extractor and reviewed by a second. Any discrepancies were discussed between extractor and reviewer. A RoB assessment was performed for each included study using an adapted Newcastle–Ottawa scale for cross-sectional studies (see Supplementary Appendix II). The following criteria were assessed: representativeness, sample size, and use of verified laboratory methods.

### Data and Meta-analysis

HIV subtype prevalence data were grouped at the global, regional, and country levels and across key populations or study period (time trends). Data were summarized in a descriptive and weighted analysis. The study period was categorized as 2010–2015 and 2016–2021. Key populations included MSM, PWIDs, sex workers, and prison inmates. The pooled analyses on the regional level were based on a meta-analysis using a random-effects model of observed proportions. The observed proportions were transformed using the Freeman–Tukey double arcsine transformation and pooled using the DerSimonian and Laird estimator. For the pooled global analysis, regional estimates were weighted according to the number of people living with HIV, regionally and according to Joint United Nations Programme on HIV/AIDS (UNAIDS) estimates (2022). For the global estimates, the meta-analysis was based on untransformed proportions to allow the derivation of the variance for the weighted global estimate. The sensitivity analyses used the same estimation procedures; however, when certain subtype counts were not reported but were deduced as 0 (ie, when all subjects were accounted for), the counts were imputed accordingly. To test for heterogeneity (*I*^2^), a subanalysis was conducted by RoB (low RoB vs some concern/high RoB). All analyses were performed using R (version 4.1.0) and the metafor [22] and forestplot packages (see Supplementary Appendix I for more details).

## RESULTS

A total of 454 studies with an overall sample size of approximately 610 000 across 91 countries were included in the SLR, with 1 large study from the United States (sample size close to 328 000 included in the analysis). Table 1 summarizes the included study characteristics; most studies were cross-sectional in design (n = 413 [91%]) where 281 studies (62%) had a sample size between 50 and 200, and 383 studies (84%) had a study length of 3 years or less and used a convenience sampling method (n = 387 [85%]).

**Table 1.** Included Study Characteristics (N = 454)

Characteristic	No. of Studies (%)
United Nations region	
Asia and the Pacific	157 (34.6)
Eastern Europe and Central Asia	17 (3.7)
Western and Central Europe and North America	90 (19.8)
Middle East and North Africa	20 (4.4)
Western and Central Africa	58 (12.8)
Eastern and Southern Africa	53 (11.7)
Latin America and the Caribbean	45 (9.9)
Multiple regions^a^	14 (3.2)
Study design	
Pooled cross-sectional	2 (0.4)
Case control	3 (0.7)
Longitudinal (baseline characteristics of a cohort)	36 (7.9)
Cross-sectional	413 (91.0)
Sample size	
<50	89 (19.6)
50–199	281 (61.9)
≥200	84 (18.5)
Study length, y	
<1	120 (26.4)
1	134 (29.5)
2	77 (17.0)
3	52 (11.5)
4	16 (3.5)
5	18 (4.0)
6	22 (4.8)
7	7 (1.5)
8	5 (1.1)
10	3 (0.7)
Key populations	No. of reports (n = 171)^b^
Prison inmates	2
Sex workers	8
Heterosexual	37
People who inject drugs	50
Men who have sex with men	70
Other	4

^a^Defined as papers reporting across multiple regions (not included in analysis): 101 studies from China, 13 studies from Russia, 15 studies from the United States, 15 studies from Cameroon, 15 studies from South Africa, 33 studies from Brazil.

^b^There was a total of 104 papers reporting subtypes across key populations, with 171 reports overall as some papers reported on >1 group. While there were more papers that contained key population data in their cohort, they were not reported on separately and thus not included in this count.

### HIV-2 and Coinfections

Of the studies that reported HIV-2 or HIV-1/HIV-2 coinfections, few provided quantitative information on population size, complicating prevalence estimates. Among those that did report total population, 8 studies across 8 countries (Cabo Verde, Burkina Faso, Cote d’Ivoire, Guinea-Bissau, Mali, Gabon, Cameroon, France) resulted in an estimated HIV-2 prevalence of 3% (1053 of 33 941 HIV-infected individuals). For HIV-1/HIV-2 coinfections, 5 studies across 6 countries (Guinea-Bissau, Burkina Faso, Cote d’Ivoire, Mali, Gabon, South Africa) resulted in an estimated prevalence of 10% (338 of 3491 HIV-infected individuals). No further analysis could be conducted due to a lack of data.

### Geographic Results

**Figure 1.**
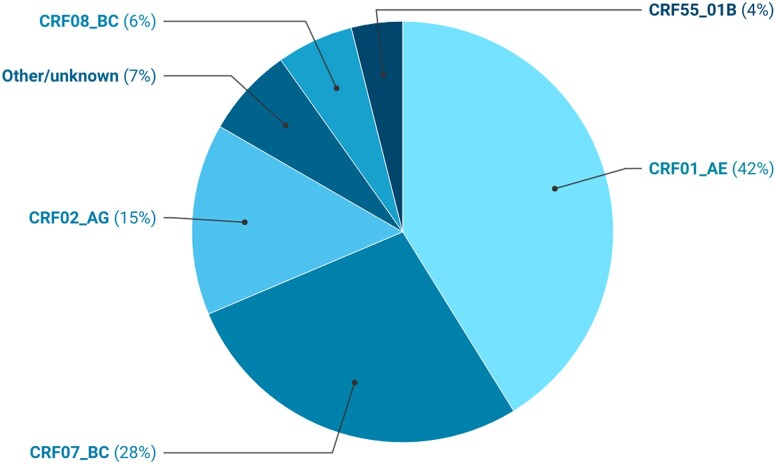
Unweighted proportion (%) of HIV global circulating recombinant forms (2010–2021).

**Figure 2.**
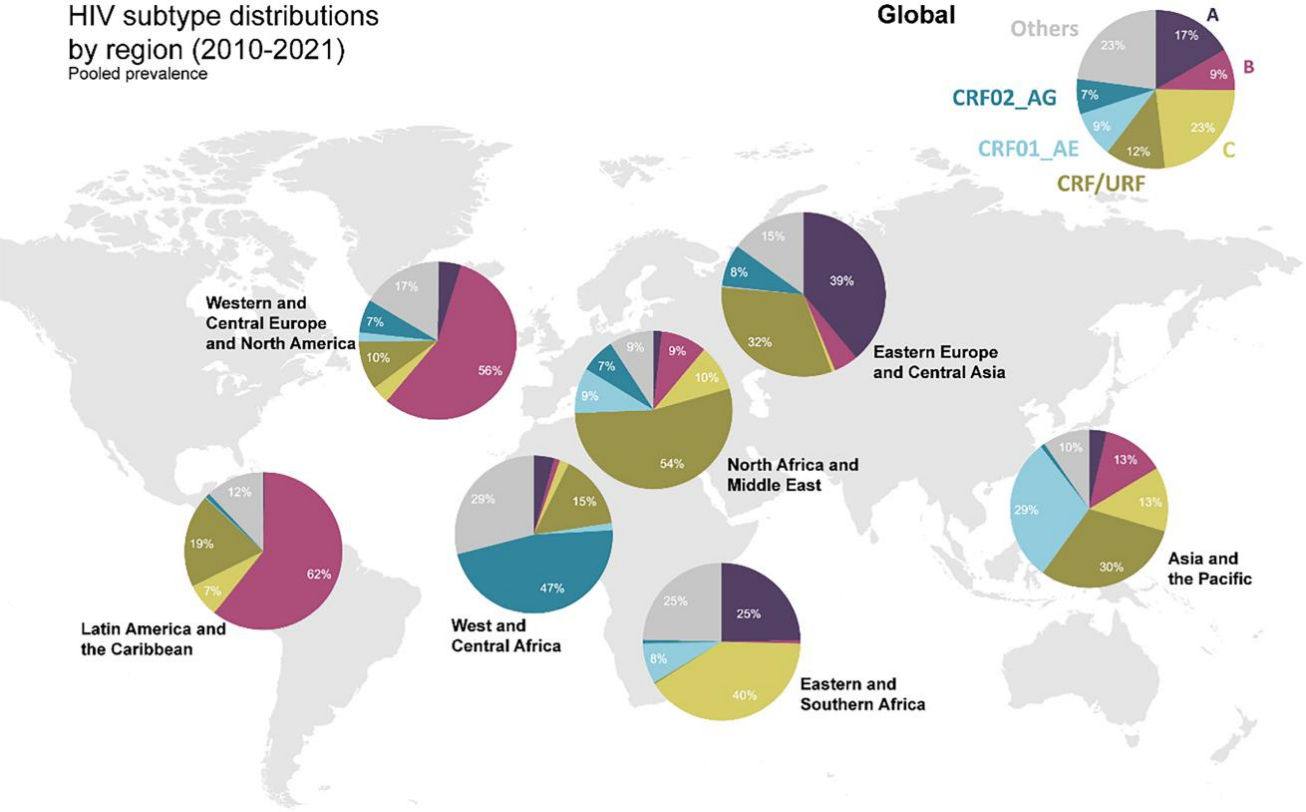
Global map of weighted HIV-1 subtype distributions (2010–2021). Others = subtypes D, F, G, H, J, other/unknown (as reported by authors); circulating recombinant form (CRF)/unique recombinant form (URF) does not include CRF01_AE or CRF02_AG since these are presented separately.

The 3 most prevalent HIV subtypes in people living with HIV worldwide are C (23.0% [95% confidence interval {CI}, 19.5%–26.5%]), followed by subtype A (16.7% [95% CI, 14.3%–19.1%]), then CRF01_AE (9.5% [95% CI, .0–19.9%]). Combined, CRFs and URFs account for 29% of all circulating subtypes. Among CRFs, CRF01_AE makes up 42% of all global CRFs, followed by CRF07_BC (28%) (Figure 1). Of note, the “other/unknown” category also contains many recombinant forms but were not reported as such by the authors and, thus, have not been considered in the CRF/URF combined prevalence estimates. Regionally, CRFs predominate in West and Central Africa (CRF02_AG), the Middle East and North Africa (MENA) (other CRFs), and Asia and the Pacific (CRF01_AE) (Figure 2). Subtype B was primarily found in Western and Central Europe and North America (WCE/NA) and Latin America and the Caribbean (LATAM). Subtype C was reported primarily in Eastern and Southern Africa, while subtype A was most reported in Eastern Europe and Central Asia (Figure 2; Table 2). Few papers reported on subtypes F, G, H, and J; thus, sample sizes are small and caution should be given when drawing conclusions from these estimates. A final table on unweighted subtypes reported by country can be found in the Supplementary Appendix 3, Table 5.

**Table 2.** HIV-1 Subtype Prevalence by Joint United Nations Programme on HIV/AIDS Region and Globally (2010–2021)

Region	A	B	C	Other CRFs^a^	CRF01_AE	CRF02_AG	D	F	G	H	J	Other/Unknown^b^	URF
Global	16.7 (14.3–19.1)	8.5 (2.7–14.3)	23.0 (19.5–26.5)	9.2 (3.6–14.7)	9.5 (.0–19.9)	7.2 (2.9–11.5)	7.7 (6.0–9.3)	3.4 (.0–8.2)	2.3 (.4–4.1)	1.6 (.0–4.3)	0.6 (.1–1.1)	7.3 (4.4–10.1)	3.1 (.0–12.6)
Eastern and Southern Africa	24.7 (18.1–31.7)	0.7 (.1–1.5)	**40.6 (30.7–50.0)**	0.0 (.0–.7)	8.3 (.5–22.4)	0.8 (.1–2.2)	**12.5 (9.0–16.5)**	4.0 (.0–21.7)	0.9 (.1–2.2)	2.0 (.0–8.3)	NR	5.2 (3.1–7.7)	0.3 (.0–1.1)
West and Central Africa	4.2 (2.8–5.8)	1.2 (.5–2.0)	1.9 (.9–3.2)	7.1 (5.7–8.6)	1.5 (.5–2.7)	**46.9 (41.9–51.8)**	2.0 (1.3–3.0)	2.8 (1.9–3.8)	8.8 (6.2–11.8)	1.3 (.5–2.5)	5.1 (.0–23.0)	9.0 (4.4–14.9)	8.2 (4.5–12.8)
Middle East and North Africa	1.7 (.5–3.3)	9.4 (2.9–18.4)	9.5 (2.2–20.2)	**49.9 (32.8–62.4)**	9.3 (1.5–21.3)	7.1 (2.6–13.4)	2.8 (.0–12.0)	0.3 (.0–1.5)	2.4 (.4–5.4)	NR	NR	3.7 (.1–10.9)	3.8 (1.9–6.3)
Asia and the Pacific	3.6 (.6–8.5)	12.8 (8.8–17.2)	13.2 (6.2–22.0)	24.0 (20.4–27.8)	**29.5 (25.4–33.6)**	0.9 (.3–1.8)	0.8 (.0–3.2)	0.0 (.0–.1)	0.8 (.0–2.7)	NR	NR	8.0 (5.1–11.4)	6.4 (3.7–9.6)
Latin America and the Caribbean	0.2 (.0–1.2)	**60.5 (53.9–66.5)**	7.0 (3.8–10.9)	11.8 (1.9–27.6)	0.2 (.0–.6)	0.9 (.0–4.0)	0.1 (.0–.3)	**5.8 (4.1–7.7)**	0.7 (.1–1.8)	NR	1.1 (.0–4.8)	4.4 (1.6–8.2)	7.3 (3.5–12.1)
Eastern Europe and Central Asia	**38.9 (21.2–55.2)**	4.8 (2.3–8.2)	0.7 (.2–1.5)	28.8 (6.2–54.4)	0.3 (.0–1.1)	8.3 (.0–38.1)	NR	NR	2.2 (.1–6.1)	NR	NR	12.8 (.0–44.5)	3.1 (1.7–4.7)
Western and Central Europe and North America	4.6 (3.0–6.5)	**56.4 (50.5–62.1)**	3.6 (2.3–5.0)	6.7 (2.3–13.1)	1.9 (1.2–2.7)	6.8 (4.8–9.2)	0.8 (.3–1.4)	4.5 (2.5–7.1)	1.3 (.8–2.0)	0.1 (.0–.5)	0.1 (.0–0.4)	9.9 (5.3–15.7)	3.3 (1.1–6.6)

Data are presented as percentage (95% confidence interval). Bolded values are to highlight the largest prevalence(s) of each subtype across regions.

Abbreviations: CRF, circulating recombinant form; NR, not reported; URF, unique recombinant form.

^a^Other CRFs = any reported CRFs other than CRF01_AE and CRF02_AG.

^b^Other/unknown = subtypes reported by authors as such.

### Key Populations

A total of 104 of 454 studies reported on HIV subtype prevalence across key populations, with some studies reporting on multiple groups for a total of 171 reports and an overall sample size of approximately 35 000. Most reporting focused on MSM (41%), followed by PWID (29%), then heterosexual persons (22%); few studies reported on sex workers, prison inmates, and others (elderly male clients of sex workers, bisexual individuals, and sexual contact not defined further; data not shown). Among MSM, subtype B accounted for around 21% (95% CI, 19.2%–23.4%) of all HIV infections, while CRFs (other CRFs, CRF01_AE and CRF02_AG) made up 57%, globally. For PWIDs, 28% (95% CI, 11.4%–40.4%) of HIV infections was subtype A. Among heterosexual persons, CRFs accounted for around 40% (other CRFs, CRF01_AE and CRF02_AG) of all reported HIV infections, globally (Table 3).

**Table 3.** Weighted HIV-1 Subtype Prevalence by Key Populations, Globally, 2010–2021

Population	A	B	C	Other CRFs^a^	CRF01_AE	CRF02_AG	Other/Unknown^b^	URF
MSM	3.0 (2.0–4.0)	21.3 (19.2–23.4)	0.9 (.3–1.4)	15.0 (12.4–17.6)	18.5 (16.3–20.8)	**23.5 (19.2–27.7)**	4.7 (3.2–6.2)	5.3 (4.1–6.6)
HTX	7.0 (.0–36.5)	10.4 (5.3–15.4)	**18.6 (10.5–26.7)**	16.8 (11.2–22.3)	19.8 (12.0–27.6)	4.5 (.0–22.3)	14.2 (8.4–20.0)	**8.7 (3.1–14.4)**
PWID	**28.0 (11.4–40.4)**	10.4 (4.4–16.3)	3.8 (.0–19.8)	18.8 (13.7–23.8)	7.6 (2.8–12.4)	6.3 (.0–13.0)	7.1 (2.9–11.2)	4.9 (1.9–7.9)
Sex workers	8.6 (.0–45.7)	2.3 (.6–4.7)	5.6 (3.2–8.6)	16.6 (9.4–24.8)	**41.3 (28.4–50.8)**	NR	**21.0 (18.8–23.1)**	3.2 (1.2–6.0)
Prison inmates	NR	**85.5 (69.5–90.1)**	1.7 (.0–7.0)	NR	NR	6.4 (.0–25.4)	NR	NR

Data are presented as percentage (95% confidence interval). Bolded values are to highlight the largest prevalence(s) of each subtype across key populations.

Abbreviations: CRF, circulating recombinant form; HTX, heterosexual persons; MSM, men who have sex with men; NR, not reported; PWID, people who inject drugs; URF, unique recombinant form.

^a^Other CRFs = any reported CRFs other than CRF01_AE and CRF02_AG.

^b^Other/unknown = subtypes reported by authors as such. Subtypes D, F, G, H, and J were removed from this table due to limited data.

### Time Trends

HIV subtypes were stratified regionally across 2 time periods (2010–2015 and 2016–2021). In cases where subtype data were not reported annually, study start date was used instead, where the first year of the corresponding period was used as the year in the analysis. Additionally, to properly understand granular changes, studies were not included if they were 3 years or longer in length with no annual trends reported. In total, 399 papers were eligible for the analysis, with 77% in the period 2010–2015.

**Figure 3.**
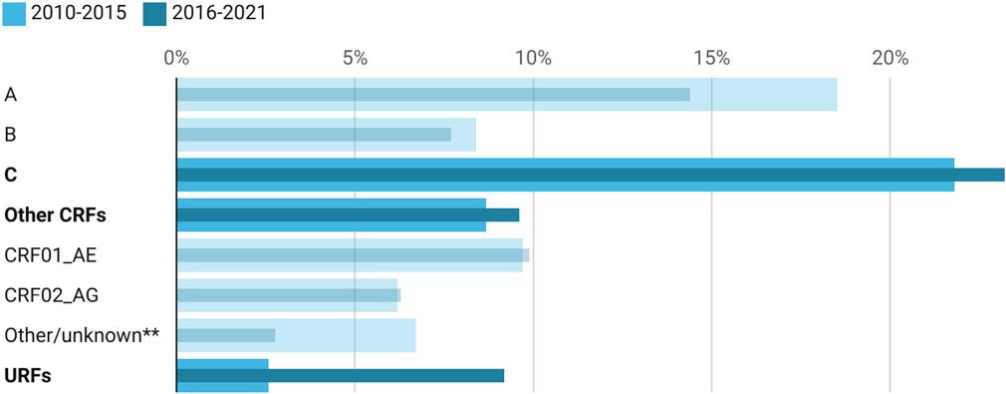
Global proportion of HIV-1 subtypes across time (2010–2021). *Other CRFs = any reported circulating recombinant forms other than CRF01_AE and CRF02_AG. **Other/unknown = subtypes reported by authors as such. Abbreviations: CRF, circulating recombinant form; URF, unique recombinant form.

Figure 3 presents the global pooled prevalence time trends of major subtypes and excludes D, F, G, H, and J due to a lack of sensitivity from few papers reporting on these subtypes. From 2010 to 2021, the global proportion of subtype C and other CRFs/URFs increased while subtype A and other/unknown subtypes decreased in prevalence. Regionally, URFs increased in West and Central Africa by around 10% and slightly in Asia and the Pacific by 5%, while decreasing slightly in LATAM (Supplementary Appendix III, Supplementary Table 4). In the MENA region, there was a 5% decrease in CRF02_AG prevalence, but other CRFs increased by the same increment. Similarly, in Asia and the Pacific, other CRFs increased by 11%, whereas CRF01_AE prevalence decreased by around 5%. Finally, for other CRFs, there was a large increase in reporting in LATAM by around 25%—the largest difference among all time trends—while also increasing by 12% in WCE/NA. There was a notable increase in subtype C in Eastern Africa and Southern Africa by 9% and the MENA region by around 6%, while decreasing by 9% in Asia and the Pacific. In WCE/NA, there was a 14% increase in subtype B prevalence while decreasing in LATAM by 15%, MENA by 10%, and Asia and the Pacific by 5%.

## DISCUSSION

This study is, to our knowledge, the first meta-analysis on the global distribution of HIV subtypes spanning the past 10 years. This large meta-analysis of approximately 610 000 participants derived from 454 published studies across 91 countries allowed us to produce comprehensive estimates of various HIV subtype prevalences. Globally, CRF/URFs accounted for 29% of all circulating HIV-1, followed by subtypes C (23%) and A (17%). Most of the 171 reports on subtype breakdowns by key populations focused on MSM and PWIDs, where CRF/URFs were overrepresented at 62% and 38%, respectively. Subtype trends over time have not changed notably from 2010 to 2021 and generally follow those seen elsewhere [4, 6], with the exception of CRF/URFs, which increased by around 8%. Finally, HIV-2 remains an underexplored area requiring further focus to accurately measure its prevalence as few studies reported on this.

The prominence of these subtypes within our study at global level is, in general, concordant with the ones reported by Hemelaar and colleagues' review, although their ranking differs. Their study examined HIV-1 subtype prevalence between 1990 and 2015 across 116 countries. For the period 2010–2015, Hemelaar et al reported a higher prevalence of subtype C and lower prevalence of other CRFs and subtype A compared to our review. In congruence with our findings, Bbosa et al reported an increase in CRF prevalence, reflecting the emergence of a trend since 2010. While the 4 most prevalent subtypes remain the same in our study, subtype C differs significantly in degree of prevalence across papers; this may be due to differences in scaling and dynamic changes in CRF prevalence over time.

At the regional level, Bbosa et al and Hemelaar et al reported subtype A to be most prevalent in East Africa and Russia, and some parts of Eastern Europe, while subtype B was most prevalent in Europe, the Americas, and Oceania—similar to our findings, where applicable. In both their studies, subtype C was most prevalent in Southern Africa and India, while CRFs were found in high proportions in Asia and West Africa—CRF01_AE and CRF02_AG, respectively. Our results complement their findings, except for other CRFs/URFs that were higher in prevalence in Asia and the Pacific, LATAM, and Eastern Europe/Central Asia than reported by Hemelaar et al. Overall, the increasing prevalence of CRFs within Asia and LATAM is concerning and requires further investigation.

### Strengths and Limitations

This review was conducted by searching 3 primary literature databases, resulting in a large number of pooled participants across geographically diverse regions. As our search criteria included any language, we got many non-English-language papers, which we were able to translate in-house. However, the Chinese papers needed to be outsourced and required extensive resources. Thus, we had to prioritize papers from less reported areas (to increase representativeness in China) and those from more recent years since the aim of the SLR was to have an updated and as-recent-as-possible understanding of subtype trends. Indeed, selecting more recent papers would have a small effect on the results, but as there were around 100 papers from China overall, this selection represents just 10% of those. Additionally, while it would have been ideal to supplement the literature review with datasets from regional- and national-level databases, it was beyond the scope of our resources. Yet, as these databases are typically based on routine surveillance data, subpopulations at higher risk of HIV infection may be underrepresented, which may be better captured in the literature.

Interpretation of this study warrants caution and only within the context of certain limitations. First, most studies obtained a low score for representativeness as their data were collected using convenience sampling with varying sample sizes. These factors could have led to either an under- or overestimation of subtype prevalence. Second, there are many small-sized studies, resulting in large CIs, which in turn disallows us from making appropriate inferences. However, upon conducting a RoB subanalysis, most subtype prevalence remained similar, yet subtype C increased by 7% when only including papers with a low RoB.

Third, as is the case in most meta-analyses on burden of disease, a high heterogeneity was observed between studies. An important source of heterogeneity may be related to the array of laboratory methods, reference databases, and genome sequences used to identify HIV subtypes (data not shown), which as a corollary may impact the reporting of subtypes, both at intra- and interregional levels.

Fourth, not all geographic regions and countries were represented equally, with some countries having only 1 small-sized study or none at all, which may preclude us from drawing regional comparisons. For example, in China alone we included >100 papers—significantly more than any other country—making it overrepresentative of the Asia-Pacific region. Another element to consider with global estimates is that most participants included in the review came from China and the United States. To minimize these effects, we weighted the results against UNAIDS estimates of people with HIV at the regional level. Finally, the number of captured papers reporting on subtype prevalence changed over time, where there were more during the first period (2010–2015) than in the second (2016–2021).

Finally, although we were interested in the circulation of HIV subtypes from 2010 onward, the year of data collection does not always equate to the year of diagnosis, meaning that our results may have included subtypes circulating before this cutoff date.

Despite these limitations, it is noteworthy that the high heterogeneity due to inclusion of studies from different settings, including family planning clinics, HIV clinics, and community-based settings, has enabled us to capture a more representative HIV-infected population across the world.

### Epidemiological Research Gaps

There is a lack of data on HIV-2 infections, and current surveillance and diagnostic testing are inadequate in assessing HIV-2 burden and genetic diversity. The development of point-of-care testing to detect and distinguish between HIV-1 and HIV-2 infection is urgently needed, along with larger reference datasets, such as within Los Alamos HIV database, to elucidate the genetic diversity pertaining to HIV-2 subtypes.

Another key epidemiological gap is the high possibility of mis/underreporting of key populations and transmission events by behavior, which may be more marked in certain contexts. As a result, estimates of subtypes may be less accurate and comparisons between different geographical regions hampered. It is noteworthy that as of December 2020, 69 countries, more than half of which are in Africa and the Middle East, still criminalize same-sex relationships [23]. Indeed, in the reporting of sexual activity among participants in included studies, stigma and criminalization may have led to underreporting of MSM and other behaviors that increase a person's risk of HIV infection. Even after 4 decades of HIV research, such issues continue to bias results, particularly in certain countries. Future research using respondent-driven sampling could produce a more representative sample than more commonly used convenience sampling methods.

As the HIV picture changes, so too should our tools and surveillance—through improved subtyping methods that are easily accessible and affordable, more diverse reference databases, and standardized reporting across studies. Furthermore, the lack of sampling method leading to a high to medium RoB in many studies calls into focus the need for sampling methods that are representative of the general and key populations.

## CONCLUSIONS

HIV and its subsequent prevention and surveillance continue to pose major challenges to reducing the burden of disease around the world. Future research should focus on identifying geographic trends in sampling studies over time, and how the use of various subtyping tools affects observed prevalence. Among the hurdles facing HIV vaccine development is the global HIV-1 genetic diversity, where recombinants (CRFs and URFs) now constitute the highest proportion of circulating HIV while continuing to increase in prevalence. Ideally, a globally effective HIV vaccine will need to prevent the most prevalent subtypes and recombinants [24]. Given the dynamic nature of HIV-1, coupled with the continuous migration of populations and increases in international transmission events, similar meta-analyses should be periodically carried out.

## Supplementary Data

Supplementary materials are available at *The Journal of Infectious Diseases* online. Consisting of data provided by the authors to benefit the reader, the posted materials are not copyedited and are the sole responsibility of the authors, so questions or comments should be addressed to the corresponding author.

## Supplementary Material

jiad327_Supplementary_Data

## Notes

***Acknowledgements.*** The authors thank all contributors to the literature review, all researchers who have provided information and data allowing the conduct of the meta-analysis presented as part of this manuscript, and the staff of P95 and/or Janssen and J&J who have contributed. We specifically thank: Gonzalo Bello Bentancor (Lab de AIDS e Imunologia Molecular, Instituto Oswaldo Cruz, FIOCRUZ, Rio de Janeiro, Brazil), Marta Bes (Banc de Sang i Teixits, Barcelona, Spain), Servet Uluer Biçeroğlu (Ege University Hospital, Bornova-Izmir-Turkey), Roxana Cernat (University “Ovidius” of Constanta, Constanța, Romania), Wassim Chehadeh (Kuwait University, Faculty of Medicine, Department of Microbiology), Carmen María González Domenech (University of Malaga, Malaga, Spain), Joseph Fokam (University of Buea, Buea, Cameroon), Tiago Gräf (Instituto Carlos Chagas, Fundação Oswaldo Cruz, Curitiba, Brazil), Martin Hoenigl (University of California at San Diego, San Diego, California), Oliver Laeyendecker (Johns Hopkins University, Baltimore, Maryland), Kledoaldo Lima (Federal University of Pernambuco, Brazil), Mor Orna (Tel Aviv University, Tel Aviv, Israel), Kara Osbak (Institute of Tropical Medicine Antwerp, Antwerp, Belgium), Nikos Pantazis and Dimitrios Paraskevis (National and Kapodistrian University of Athens, Athens, Greece), Art Poon (Western University, Ontario, Canada), Matthew Price (University of California, San Francisco), Sonia Mara Raboni (Universidade Federal do Paraná, Brazil), Barbara Rossetti (Infectious Diseases Unit, Azienda Ospedaliero-Universitaria Senese, Siena, Italy), Karol Serwin (Pomeranian Medical University, Szczecin, Poland), Maja Stanojevic (University of Belgrade Faculty of Medicine, Institute of Microbiology and Immunology), and Yong-Tang Zheng (Kunming Institute of Zoology, Chinese Academy of Sciences, Yunnan, China). From the Medical Research Council/Uganda, we thank Sylvia Kiwuwa-Muyingo. From P95, we thank Zuleika Aponte, Marc Baay, Robin Bruyndonckx, German Cantillo-Mackenzie, Luisa Carbal, Laura Mora, Ingrid Tatiana Sepúlveda Pachón, Margarita Riera, and Thao Mai Phuong Tran for their roles in various phases of the project. We thank Ana Goios for her creative design of the map figure. From Janssen, we thank Rachael DiSantotefano, Meagan Rubel, Daniel Stieh, and Widagdo for contributing their expertise to the development of this project and manuscript.

***Data availability*.** The full list of 454 included references can be provided upon request.

***Financial support*.** This study was funded by Janssen and conducted in collaboration with P95, the Medical Research Council/Uganda, and the Kenya Medical Research Institute. Funding to pay the Open Access publication charges for this article was provided by Janssen.
