# Supplementary material for: Geographic and Population Distributions of Human Immunodeficiency Virus (HIV)–1 and HIV-2 Circulating Subtypes: A Systematic Literature Review and Meta-analysis (2010–2021)
Source: J Infect Dis. 2023 Aug 18;228(11):1583–91. doi: 10.1093/infdis/jiad327 (PMC10681860; doi:10.1093/infdis/jiad327)
Supplement: jiad327_Supplementary_Data [file jiad327_supplementary_data.docx]

Supplementary Info

1. Appendix I
   1. Detailed Methods and PRISMA

We started building the search strategy using the one described by Hemelaar et al (1). In short, they used terms related to “HIV,” “subtype,” “recombinant,” and “epidemiology.” The search of Hemelaar et al. produced 37,938 de-duplicated references, from which 2,029 full text articles were screened for eligibility. In total, 820 studies were included in the review. Given that ~2% of the references were used in the final review, we have optimized the search string to reduce the number of overall hits while retaining relevance using an online open-source tool called ‘[searchrefiner](https://ielab.io/searchrefiner)’ (2). The steps for using this tool can be found [here](https://sr-accelerator.com/#/help/searchRefiner). This tool was used to check search words for relevance and compared against 50 seed PMIDs, in combination with Vlookup in Excel. Through an iterative process, we removed key words related to those listed above that produced many hits but did not improve relevance. The final keywords used in our study can be found in the PICO table below (Table 1):

Table 1. Keywords used in database search

| Domain | Keywords |
| --- | --- |
| Disease | HIV-1, HIV-2, HIV |
| Focus | Subtype, clade, CRF, URF, recombinant, strain, sequence, type, genotype, variant |
| Epidemiology | Epidemiology (+ molecular & genetic), incidence, prevalence, distribution, characterization, frequency, genetic diversity |
| Filters | 2010-2021 [publication date] |

The searches in PubMed, Embase, and CABI were conducted in June 2021. A total of 37,882 potentially relevant articles were identified. Deduplication was done using EndNote and 25,535 references were screened based on a priori inclusion and exclusion criteria (Figure 1. PRISMA diagram; protocol). Next, the remaining 9,405 references were imported into Rayyan (3) for title/abstract screening by two reviewers and any conflicts were resolved among reviewers or by a third reviewer if needed. At full-text screening, non-English language articles were translated using DeepL, except Chinese language papers (n=46) requiring a translation service. Ten of the most recent Chinese papers (from 2015 onwards) were selected for translation and extraction. Additionally, bibliographies of relevant review articles identified in the screening phase were hand searched and a total of 28 additional studies were identified.

For papers spanning 2010 (i.e., data collection overlapped 2010 and there was no subtype breakdown by year) or those with unclear/unreported dates of data collection, corresponding authors were contacted by email to request a data breakdown by year or date clarification. An additional 384 references could not be included due to an overlap in data with 2010 or unclear dates where we were not able to contact authors, did not hear back, or did not receive usable data; author response rate was around 8%.

At the extraction phase, data items included variables such as overall study details, patient demographics, sampling approach and laboratory methods used, recruitment setting, and HIV subtype prevalence data regionally, annually, or across key populations.

Aruba was grouped in Latin America and the Caribbean, Taiwan and Australia were grouped in Asia and the Pacific, and Puerto Rico was considered part of the Caribbean. For the forest plots (data not shown), the following code was used: @Manual{,title = {forestplot: Advanced Forest Plot Using 'grid' Graphics},author = {Max Gordon and Thomas Lumley},year = {2021},note = {R package version 2.0.1},url = { [*https://CRAN.R-project.org/package=forestplot*}](https://CRAN.R-project.org/package=forestplot%7d),}

- - Expanded PubMed search string

(("hiv 1"[MeSH Terms] OR "hiv 1"[All Fields] OR "hiv 1"[All Fields] OR ("hiv 2"[MeSH Terms] OR "hiv 2"[All Fields] OR "hiv 2"[All Fields]) OR ("hiv"[MeSH Terms] OR "hiv"[All Fields])) AND ("subtype"[All Fields] OR "subtyped"[All Fields] OR "subtypes"[All Fields] OR "subtyping"[All Fields] OR "subtypings"[All Fields] OR ("clade"[All Fields] OR "clade s"[All Fields] OR "clades"[All Fields]) OR "crf"[All Fields] OR "urf"[All Fields] OR ("f8 protein human"[Supplementary Concept] OR "f8 protein human"[All Fields] OR "recombinate"[All Fields] OR "recombinant"[All Fields] OR "recombinants"[All Fields] OR "recombinated"[All Fields] OR "recombinates"[All Fields] OR "recombination, genetic"[MeSH Terms] OR ("recombination"[All Fields] AND "genetic"[All Fields]) OR "genetic recombination"[All Fields] OR "recombination"[All Fields] OR "recombinations"[All Fields] OR "recombinational"[All Fields] OR "recombinative"[All Fields] OR "recombine"[All Fields] OR "recombined"[All Fields] OR "recombineered"[All Fields] OR "recombineering"[All Fields] OR "recombines"[All Fields] OR "recombining"[All Fields]) OR ("sprains and strains"[MeSH Terms] OR ("sprains"[All Fields] AND "strains"[All Fields]) OR "sprains and strains"[All Fields] OR "strain"[All Fields] OR "strains"[All Fields] OR "strain s"[All Fields]) OR ("base sequence"[MeSH Terms] OR ("base"[All Fields] AND "sequence"[All Fields]) OR "base sequence"[All Fields] OR "sequence"[All Fields] OR "sequences"[All Fields] OR "sequence analysis"[MeSH Terms] OR ("sequence"[All Fields] AND "analysis"[All Fields]) OR "sequence analysis"[All Fields] OR "sequencing"[All Fields] OR "sequence s"[All Fields] OR "sequenceable"[All Fields] OR "sequenced"[All Fields] OR "sequenceing"[All Fields] OR "sequencer"[All Fields] OR "sequencers"[All Fields] OR "sequencies"[All Fields] OR "sequencings"[All Fields]) OR "type"[All Fields] OR ("genotype"[MeSH Terms] OR "genotype"[All Fields] OR "genotypes"[All Fields] OR "genotypic"[All Fields] OR "genotype s"[All Fields] OR "genotyped"[All Fields] OR "genotyper"[All Fields] OR "genotypical"[All Fields] OR "genotypically"[All Fields] OR "genotyping"[All Fields] OR "genotypings"[All Fields] OR "genotypization"[All Fields]) OR ("variant"[All Fields] OR "variant s"[All Fields] OR "variants"[All Fields])) AND ("epidemiologies"[All Fields] OR "epidemiology"[MeSH Subheading] OR "epidemiology"[All Fields] OR "epidemiology"[MeSH Terms] OR "epidemiology s"[All Fields] OR ("epidemiology"[MeSH Subheading] OR "epidemiology"[All Fields] OR "incidence"[All Fields] OR "incidence"[MeSH Terms] OR "incidences"[All Fields] OR "incident"[All Fields] OR "incidents"[All Fields]) OR ("epidemiology"[MeSH Subheading] OR "epidemiology"[All Fields] OR "prevalence"[All Fields] OR "prevalence"[MeSH Terms] OR "prevalance"[All Fields] OR "prevalences"[All Fields] OR "prevalence s"[All Fields] OR "prevalent"[All Fields] OR "prevalently"[All Fields] OR "prevalents"[All Fields]) OR ("distribute"[All Fields] OR "distributed"[All Fields] OR "distributer"[All Fields] OR "distributers"[All Fields] OR "distributes"[All Fields] OR "distributing"[All Fields] OR "distributional"[All Fields] OR "distributions"[All Fields] OR "supply and distribution"[MeSH Subheading] OR ("supply"[All Fields] AND "distribution"[All Fields]) OR "supply and distribution"[All Fields] OR "distribution"[All Fields]) OR ("molecular epidemiology"[MeSH Terms] OR ("molecular"[All Fields] AND "epidemiology"[All Fields]) OR "molecular epidemiology"[All Fields]) OR ("molecular epidemiology"[MeSH Terms] OR ("molecular"[All Fields] AND "epidemiology"[All Fields]) OR "molecular epidemiology"[All Fields] OR ("genetic"[All Fields] AND "epidemiology"[All Fields]) OR "genetic epidemiology"[All Fields]) OR ("epidemiology"[MeSH Subheading] OR "epidemiology"[All Fields] OR "frequency"[All Fields] OR "epidemiology"[MeSH Terms] OR "frequence"[All Fields] OR "frequences"[All Fields] OR "frequencies"[All Fields]) OR ("genetic variation"[MeSH Terms] OR ("genetic"[All Fields] AND "variation"[All Fields]) OR "genetic variation"[All Fields] OR ("genetic"[All Fields] AND "diversity"[All Fields]) OR "genetic diversity"[All Fields]))) AND (2010:2021[pdat])

Translations

HIV-1: "hiv-1"[MeSH Terms] OR "hiv-1"[All Fields] OR "hiv 1"[All Fields]

HIV-2: "hiv-2"[MeSH Terms] OR "hiv-2"[All Fields] OR "hiv 2"[All Fields]

HIV: "hiv"[MeSH Terms] OR "hiv"[All Fields]

subtype: "subtype"[All Fields] OR "subtyped"[All Fields] OR "subtypes"[All Fields] OR "subtyping"[All Fields] OR "subtypings"[All Fields]

clade: "clade"[All Fields] OR "clade's"[All Fields] OR "clades"[All Fields]

recombinant: "F8 protein, human"[Supplementary Concept] OR "F8 protein, human"[All Fields] OR "recombinate"[All Fields] OR "recombinant"[All Fields] OR "recombinants"[All Fields] OR "recombinated"[All Fields] OR "recombinates"[All Fields] OR "recombination, genetic"[MeSH Terms] OR ("recombination"[All Fields] AND "genetic"[All Fields]) OR "genetic recombination"[All Fields] OR "recombination"[All Fields] OR "recombinations"[All Fields] OR "recombinational"[All Fields] OR "recombinative"[All Fields] OR "recombine"[All Fields] OR "recombined"[All Fields] OR "recombineered"[All Fields] OR "recombineering"[All Fields] OR "recombines"[All Fields] OR "recombining"[All Fields]

strain: "sprains and strains"[MeSH Terms] OR ("sprains"[All Fields] AND "strains"[All Fields]) OR "sprains and strains"[All Fields] OR "strain"[All Fields] OR "strains"[All Fields] OR "strain's"[All Fields]

sequence: "base sequence"[MeSH Terms] OR ("base"[All Fields] AND "sequence"[All Fields]) OR "base sequence"[All Fields] OR "sequence"[All Fields] OR "sequences"[All Fields] OR "sequence analysis"[MeSH Terms] OR ("sequence"[All Fields] AND "analysis"[All Fields]) OR "sequence analysis"[All Fields] OR "sequencing"[All Fields] OR "sequence's"[All Fields] OR "sequenceable"[All Fields] OR "sequenced"[All Fields] OR "sequenceing"[All Fields] OR "sequencer"[All Fields] OR "sequencers"[All Fields] OR "sequencies"[All Fields] OR "sequencings"[All Fields]

genotype: "genotype"[MeSH Terms] OR "genotype"[All Fields] OR "genotypes"[All Fields] OR "genotypic"[All Fields] OR "genotype's"[All Fields] OR "genotyped"[All Fields] OR "genotyper"[All Fields] OR "genotypical"[All Fields] OR "genotypically"[All Fields] OR "genotyping"[All Fields] OR "genotypings"[All Fields] OR "genotypization"[All Fields]

variant: "variant"[All Fields] OR "variant's"[All Fields] OR "variants"[All Fields]

epidemiology: "epidemiologies"[All Fields] OR "epidemiology"[Subheading] OR "epidemiology"[All Fields] OR "epidemiology"[MeSH Terms] OR "epidemiology's"[All Fields]

incidence: "epidemiology"[Subheading] OR "epidemiology"[All Fields] OR "incidence"[All Fields] OR "incidence"[MeSH Terms] OR "incidences"[All Fields] OR "incident"[All Fields] OR "incidents"[All Fields]

prevalence: "epidemiology"[Subheading] OR "epidemiology"[All Fields] OR "prevalence"[All Fields] OR "prevalence"[MeSH Terms] OR "prevalance"[All Fields] OR "prevalences"[All Fields] OR "prevalence's"[All Fields] OR "prevalent"[All Fields] OR "prevalently"[All Fields] OR "prevalents"[All Fields]

distribution: "distribute"[All Fields] OR "distributed"[All Fields] OR "distributer"[All Fields] OR "distributers"[All Fields] OR "distributes"[All Fields] OR "distributing"[All Fields] OR "distributional"[All Fields] OR "distributions"[All Fields] OR "supply and distribution"[Subheading] OR ("supply"[All Fields] AND "distribution"[All Fields]) OR "supply and distribution"[All Fields] OR "distribution"[All Fields]

molecular epidemiology: "molecular epidemiology"[MeSH Terms] OR ("molecular"[All Fields] AND "epidemiology"[All Fields]) OR "molecular epidemiology"[All Fields]

genetic epidemiology: "molecular epidemiology"[MeSH Terms] OR ("molecular"[All Fields] AND "epidemiology"[All Fields]) OR "molecular epidemiology"[All Fields] OR ("genetic"[All Fields] AND "epidemiology"[All Fields]) OR "genetic epidemiology"[All Fields]

frequency: "epidemiology"[Subheading] OR "epidemiology"[All Fields] OR "frequency"[All Fields] OR "epidemiology"[MeSH Terms] OR "frequence"[All Fields] OR "frequences"[All Fields] OR "frequencies"[All Fields]

genetic diversity: "genetic variation"[MeSH Terms] OR ("genetic"[All Fields] AND "variation"[All Fields]) OR "genetic variation"[All Fields] OR ("genetic"[All Fields] AND "diversity"[All Fields]) OR "genetic diversity"[All Fields]

- - Embase search string

No. Query Results Results Date

#2. #1 AND ('Article'/it OR 'Article in Press'/it OR 18,035 8 Jun 2021

'Review'/it)

#1. ('hiv 1'/exp OR 'hiv 1' OR 'hiv 2'/exp OR 'hiv 2' 26,177 8 Jun 2021

OR 'hiv'/exp OR hiv) AND (subtype OR 'clade'/exp

OR clade OR 'crf'/exp OR crf OR urf OR

'recombinant'/exp OR recombinant OR 'strain'/exp

OR strain OR sequence OR type OR 'genotype'/exp

OR genotype OR 'variant'/exp OR variant) AND

('epidemiology'/exp OR epidemiology OR

'incidence'/exp OR incidence OR 'prevalence'/exp

OR prevalence OR 'distribution'/exp OR

distribution OR 'molecular epidemiology'/exp OR

'molecular epidemiology' OR (molecular AND

('epidemiology'/exp OR epidemiology)) OR 'genetic

epidemiology'/exp OR 'genetic epidemiology' OR

(('genetic'/exp OR genetic) AND

('epidemiology'/exp OR epidemiology)) OR

'frequency'/exp OR frequency OR 'genetic

diversity'/exp OR 'genetic diversity' OR

(('genetic'/exp OR genetic) AND ('diversity'/exp

OR diversity))) AND [2010-2021]/py

- - CABI search string (not available)

Figure 1. PRISMA Diagram


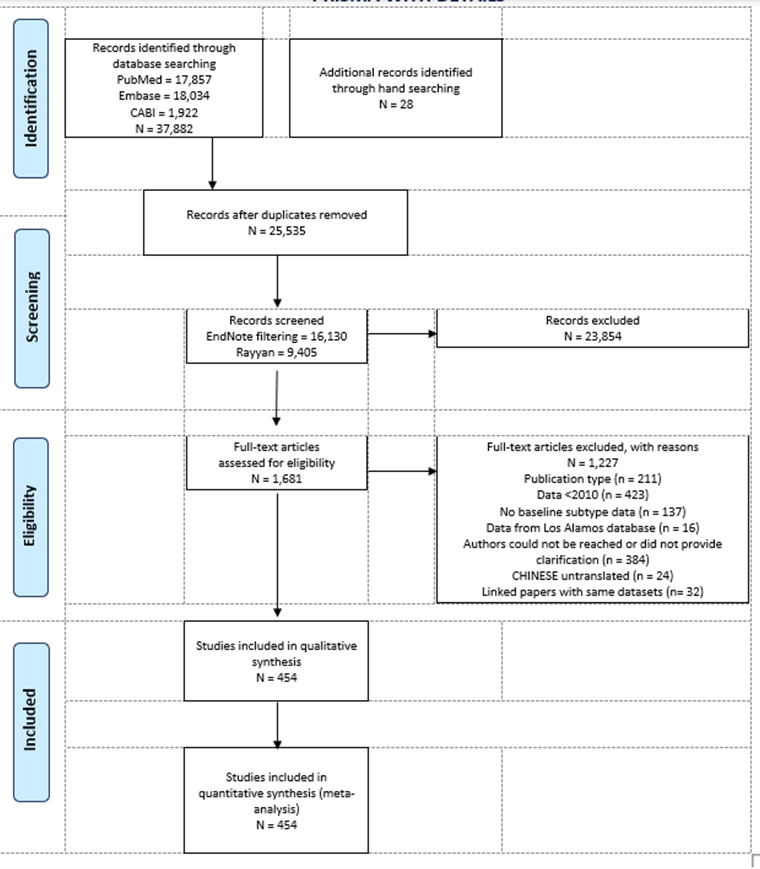


Table 2. PRISMA Checklist

| **Section and Topic** | **Item #** | **Checklist item** | **Location where item is reported** |
| --- | --- | --- | --- |
| **TITLE** | | |  |
| Title | 1 | Identify the report as a systematic review. | Lines 1-2 |
| **ABSTRACT** | | |  |
| Abstract | 2 | See the PRISMA 2020 for Abstracts checklist. | Lines 26-50 |
| **INTRODUCTION** | | |  |
| Rationale | 3 | Describe the rationale for the review in the context of existing knowledge. | Lines 86-88 |
| Objectives | 4 | Provide an explicit statement of the objective(s) or question(s) the review addresses. | Lines 91-94 |
| **METHODS** | | |  |
| Eligibility criteria | 5 | Specify the inclusion and exclusion criteria for the review and how studies were grouped for the syntheses. | Lines 104-111, 120-123 |
| Information sources | 6 | Specify all databases, registers, websites, organisations, reference lists and other sources searched or consulted to identify studies. Specify the date when each source was last searched or consulted. | Lines 103-104; Suppl. Line 13 |
| Search strategy | 7 | Present the full search strategies for all databases, registers and websites, including any filters and limits used. | Suppl. Pages 3-7 |
| Selection process | 8 | Specify the methods used to decide whether a study met the inclusion criteria of the review, including how many reviewers screened each record and each report retrieved, whether they worked independently, and if applicable, details of automation tools used in the process. | Lines 113-114  Suppl lines 13-18 |
| Data collection process | 9 | Specify the methods used to collect data from reports, including how many reviewers collected data from each report, whether they worked independently, any processes for obtaining or confirming data from study investigators, and if applicable, details of automation tools used in the process. | Lines 114-116  Suppl lines 20-22 |
| Data items | 10a | List and define all outcomes for which data were sought. Specify whether all results that were compatible with each outcome domain in each study were sought (e.g. for all measures, time points, analyses), and if not, the methods used to decide which results to collect. | Suppl lines 23-24 |
|  | 10b | List and define all other variables for which data were sought (e.g. participant and intervention characteristics, funding sources). Describe any assumptions made about any missing or unclear information. | Suppl lines 23-24 |
| Study risk of bias assessment | 11 | Specify the methods used to assess risk of bias in the included studies, including details of the tool(s) used, how many reviewers assessed each study and whether they worked independently, and if applicable, details of automation tools used in the process. | Lines 116-118  Suppl lines 128-154 |
| Effect measures | 12 | Specify for each outcome the effect measure(s) (e.g. risk ratio, mean difference) used in the synthesis or presentation of results. | Lines 120-122 |
| Synthesis methods | 13a | Describe the processes used to decide which studies were eligible for each synthesis (e.g. tabulating the study intervention characteristics and comparing against the planned groups for each synthesis (item #5)). | Suppl lines 140-146 |
|  | 13b | Describe any methods required to prepare the data for presentation or synthesis, such as handling of missing summary statistics, or data conversions. | Lines 120-133 |
|  | 13c | Describe any methods used to tabulate or visually display results of individual studies and syntheses. | Lines 120-133 |
|  | 13d | Describe any methods used to synthesize results and provide a rationale for the choice(s). If meta-analysis was performed, describe the model(s), method(s) to identify the presence and extent of statistical heterogeneity, and software package(s) used. | Lines 120-133 |
|  | 13e | Describe any methods used to explore possible causes of heterogeneity among study results (e.g. subgroup analysis, meta-regression). | Lines 132-133  Suppl lines 140-146 |
|  | 13f | Describe any sensitivity analyses conducted to assess robustness of the synthesized results. | Lines 129-131 |
| Reporting bias assessment | 14 | Describe any methods used to assess risk of bias due to missing results in a synthesis (arising from reporting biases). | NA |
| Certainty assessment | 15 | Describe any methods used to assess certainty (or confidence) in the body of evidence for an outcome. | NA |
| **RESULTS** | | |  |
| Study selection | 16a | Describe the results of the search and selection process, from the number of records identified in the search to the number of studies included in the review, ideally using a flow diagram. | Suppl lines 124-125 |
|  | 16b | Cite studies that might appear to meet the inclusion criteria, but which were excluded, and explain why they were excluded. | Overall: Suppl lines 124-125 |
| Study characteristics | 17 | Cite each included study and present its characteristics. | NR; too many studies to present individually |
| Risk of bias in studies | 18 | Present assessments of risk of bias for each included study. | NR; too many studies to present individually |
| Results of individual studies | 19 | For all outcomes, present, for each study: (a) summary statistics for each group (where appropriate) and (b) an effect estimate and its precision (e.g. confidence/credible interval), ideally using structured tables or plots. | NR; too many studies to present individually |
| Results of syntheses | 20a | For each synthesis, briefly summarise the characteristics and risk of bias among contributing studies. | Lines 140-146;  Suppl lines 129-154 |
|  | 20b | Present results of all statistical syntheses conducted. If meta-analysis was done, present for each the summary estimate and its precision (e.g. confidence/credible interval) and measures of statistical heterogeneity. If comparing groups, describe the direction of the effect. | Lines 178-179; 190-191; Suppl lines 157-158 |
|  | 20c | Present results of all investigations of possible causes of heterogeneity among study results. | Suppl lines 140-146 |
|  | 20d | Present results of all sensitivity analyses conducted to assess the robustness of the synthesized results. | NR |
| Reporting biases | 21 | Present assessments of risk of bias due to missing results (arising from reporting biases) for each synthesis assessed. | NA |
| Certainty of evidence | 22 | Present assessments of certainty (or confidence) in the body of evidence for each outcome assessed. | NA |
| **DISCUSSION** | | |  |
| Discussion | 23a | Provide a general interpretation of the results in the context of other evidence. | Lines 222-247 |
|  | 23b | Discuss any limitations of the evidence included in the review. | Lines 288-307 |
|  | 23c | Discuss any limitations of the review processes used. | Lines 249-286 |
|  | 23d | Discuss implications of the results for practice, policy, and future research. | Lines 309-317 |
| **OTHER INFORMATION** | | |  |
| Registration and protocol | 24a | Provide registration information for the review, including register name and registration number, or state that the review was not registered. | Lines 97-101 |
|  | 24b | Indicate where the review protocol can be accessed, or state that a protocol was not prepared. | Lines 97-101 |
|  | 24c | Describe and explain any amendments to information provided at registration or in the protocol. | Lines 97-101 |
| Support | 25 | Describe sources of financial or non-financial support for the review, and the role of the funders or sponsors in the review. | Lines 351-353 |
| Competing interests | 26 | Declare any competing interests of review authors. | Lines 355-359 |
| Availability of data, code and other materials | 27 | Report which of the following are publicly available and where they can be found: template data collection forms; data extracted from included studies; data used for all analyses; analytic code; any other materials used in the review. | Lines 318-319 |

1. Appendix II
   1. Risk of Bias assessment (adapted Newcastle-Ottawa scale)

In order to assess the risk of bias in each study, we used an adapted version of the Newcastle-Ottawa scale(4) for cross-sectional studies. Each study was awarded a score out of six points and assessed based on representativeness, sample size, and use of verified laboratory methods (Figure 2). Each criterion was scored with a value between 0 and 2, with higher scores indicating lower risk of bias (Table 3). Scores for each paper were summed, where 0-3 points was considered to have a high RoB and 4-6 a low RoB. The lack of randomization in the included studies resulted in 73% and 27% of studies having a high and low risk of bias, respectively (Figure 3). This meant that we were unable to extrapolate the findings to a wider population. As most of the papers reported on only one key population, it is impossible to extrapolate data from these key groups to a wider population of e.g MSM.

We also conducted a subanalysis stratifying by risk of bias as low vs some concern/high risk. No significant differences in pooled prevalence by subtype were found, except for subtypes C (low: 28.5 [CI: 23.9 - 33.2] vs some concern/high risk of bias: 21.5 [CI: 17.7 - 25.3]) and F (low: 2.0 [CI: 0.1 - 3.9] vs some concern/high risk of bias: 4.2 [CI: 0.0 - 10.1]), which data suggests is significantly different. Subtypes H and J had low I^2^ when stratified by RoB possibly due to the low number of papers and sample sizes reported. This subanalysis did not appear to reduce heterogeneity except in subtypes H and J.

Figure 2. Adapted NOS assessment


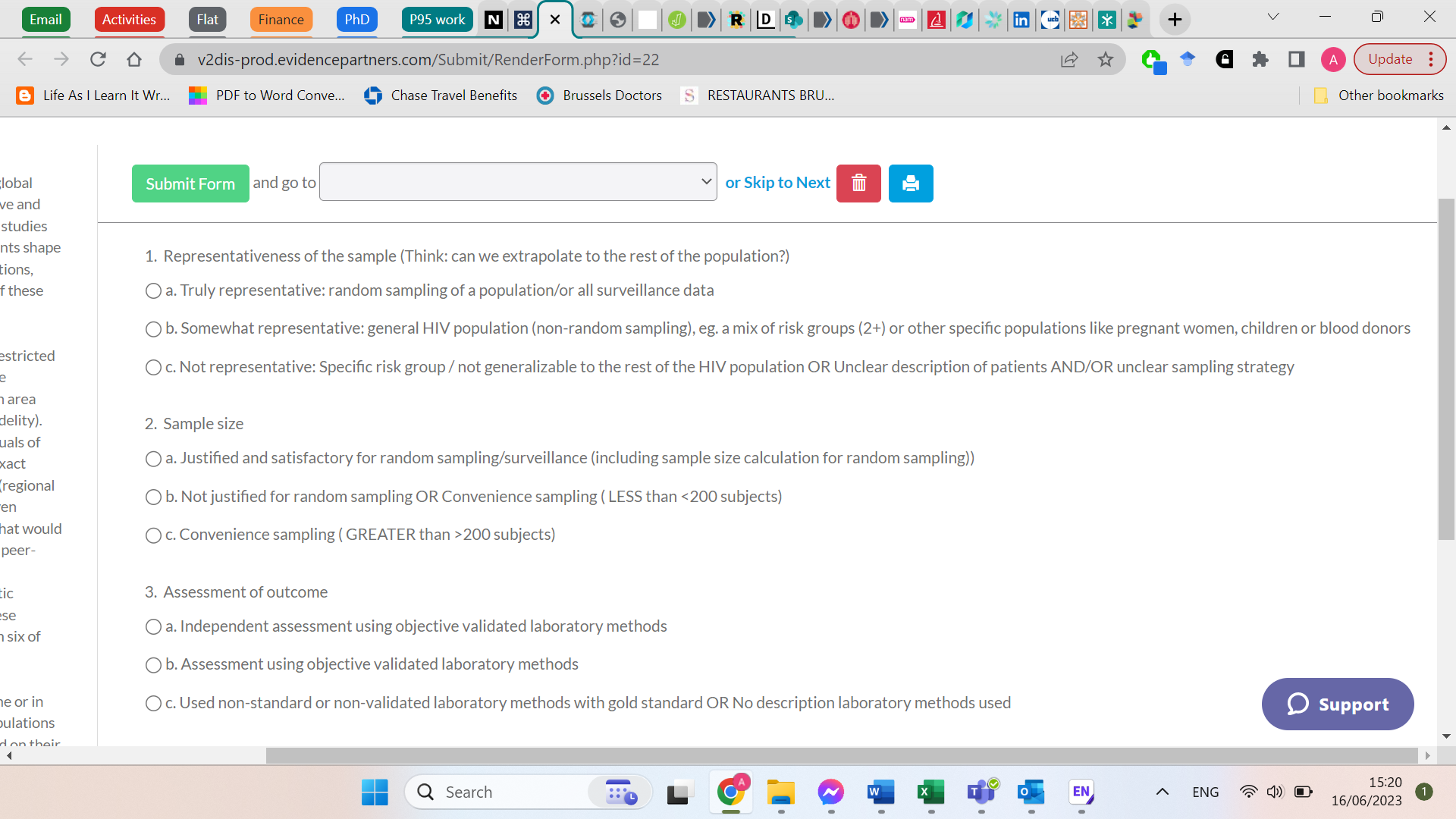


Table 3. Answer scoring per type of bias

| **Bias** | **Answer (based on Figure 2 above)** | **Score*** |
| --- | --- | --- |
| Representativeness | 1A | 2 |
|  | 1B | 1 |
|  | 1C | 0 |
| Sample size | 2A | 2 |
|  | 2B | 0 |
|  | 2C | 1 |
| Lab assessment | 3A | 2 |
|  | 3B | 2 |
|  | 3C | 1 |

* A higher score indicates a lower risk of bias

Figure 3. Risk of bias (RoB) scores (N=454 papers)

1. Appendix III

Supplementary Table 4. Global and regional weighted proportion of HIV-1 subtypes across time (2010-2021)

|  | ***A*** | ***B*** | ***C*** | ***Other CRFs**** | ***CRF01_AE*** | ***CRF02_AG*** | ***D*** | ***F*** | ***G*** | ***H*** | ***J*** | ***Other/unknown***** | ***URFs*** |
| --- | --- | --- | --- | --- | --- | --- | --- | --- | --- | --- | --- | --- | --- |
| ***Global*** | | | | | | | | | | | | | |
| *2010-2015* | 18.5 (15.3 - 21.7) | 8.4 (1.3 - 15.5) | 21.8 (16.6 - 27.1) | 8.7 (0.0 - 18.5) | 9.7 (0.0 - 19.4) | 6.2 (0.0 - 16.5) | 7.6 (5.5 - 9.7) | 0.9 (0.0 - 2.9) | 2.1 (0.0 - 5.8) | 0.7 (0.2 - 1.2) | 6.1 (0.0 - 20.0) | 6.7 (3.6 - 9.8) | 2.6 (0.0 - 8.1) |
| *2016-2021* | 14.4 (7.5 - 21.2) | 7.7 (0.0 - 25.3) | 23.2 (16.5 - 30.0) | 9.6 (3.0 - 16.3) | 9.9 (0.0 - 19.8) | 6.3 (0.0 - 15.3) | 4.8 (3.3 - 6.4) | 8.2 (2.5 - 13.8) | 1.1 (0.6 - 1.7) | 2.0 (0.0 - 5.0) | 0.7 (0.0 - 2.2) | 2.8 (0.0 - 8.0) | 9.2 (1.4 - 17.0) |
| ***Eastern and Southern Africa*** | | | | | | | | | | | | | |
| *2010-2015* | 28.0 (17.9 - 38.5) | 0.8 (0.1 - 1.9) | 40.0 (25.1 - 53.2) | 1.8 (0.1 - 4.6) | 7.8 (0.5 - 21.2) | 0.2 (0.0 - 1.0) | 13.0 (7.9 - 19.0) | 0.4 (0.0 - 1.7) | 0.9 (0.0 - 2.9) | NR | NR | 6.8 (3.0 - 11.7) | 0.2 (0.0 - 1.0) |
| *2016-2021* | 21.6 (5.8 - 41.9) | 1.2 (0.0 - 4.9) | 49.1 (25.6 - 67.7) | 0.9 (0.0 - 3.6) | NR | 1.2 (0.2 - 2.7) | 6.9 (4.6 - 9.6) | 13.0 (4.7 - 23.9) | 1.8 (0.0 - 5.1) | 2.2 (0.0 - 9.0) | NR | 2.2 (0.0 - 9.0) | NR |
| ***Western and Central Africa*** | | | | | | | | | | | | | |
| *2010-2015* | 3.5 (2.4 - 4.8) | 1.1 (0.4 - 2.2) | 1.4 (0.5 - 2.7) | 7.8 (5.8 - 10.0) | 1.3 (0.4 - 2.6) | 43.9 (38.3 - 49.5) | 1.8 (1.0 - 2.7) | 2.7 (1.8 - 3.7) | 8.1 (4.9 - 12.0) | 1.4 (0.5 - 2.5) | 8.0 (0.0 - 40.2) | 12.6 (6.1 - 20.8) | 6.3 (2.5 - 11.5) |
| *2016-2021* | 7.6 (2.7 - 14.4) | 0.8 (0.1 - 2.0) | 2.9 (1.2 - 5.3) | 5.5 (4.0 - 7.2) | 1.0 (0.0 - 3.0) | 43.5 (25.7 - 61.0) | 3.3 (1.1 - 6.6) | 3.2 (0.8 - 6.7) | 6.2 (4.1 - 8.7) | 4.6 (1.6 - 8.8) | 1.0 (0.0 - 3.0) | 4.2 (0.0 - 12.5) | 16.1 (10.1 - 23.0) |
| ***Middle East and North Africa (MENA)*** | | | | | | | | | | | | | |
| *2010-2015* | 2.6 (0.0 - 9.2) | 12.6 (0.5 - 34.4) | 6.7 (0.0 - 28.8) | 61.7 (38.3 - 73.9) | 5.3 (0.0 - 19.2) | 6.5 (4.0 - 9.4) | NR | NR | 2.8 (0.0 - 8.2) | NR | NR | 1.8 (0.4 - 3.9) | NR |
| *2016-2021* | 1.4 (0.0 - 4.3) | 2.8 (0.8 - 5.7) | 12.4 (0.0 - 46.4) | 66.2 (61.7 - 69.2) | 2.5 (0.0 - 7.2) | 1.2 (0.0 - 5.2) | 7.4 (2.6 - 14.1) | NR | 6.2 (1.8 - 12.5) | NR | NR | NR | NR |
| ***Asia and the Pacific*** | | | | | | | | | | | | | |
| *2010-2015* | 2.1 (0.2 - 5.4) | 15.3 (9.2 - 22.5) | 15.3 (5.0 - 29.1) | 22.6 (17.4 - 28.1) | 31.2 (25.9 - 36.6) | 0.8 (0.0 - 2.3) | 0.3 (0.0 - 2.1) | 0.0 (0.0 - 0.1) | 0.6 (0.0 - 3.3) | NR | NR | 7.0 (4.2 - 10.5) | 4.7 (2.5 - 7.5) |
| *2016-2021* | 7.7 (0.0 - 31.8) | 10.4 (4.9 - 17.6) | 6.2 (0.8 - 15.7) | 33.9 (26.5 - 41.5) | 26.3 (20.0 - 32.9) | 0.1 (0.0 - 0.4) | NR | NR | 0.2 (0.0 - 0.7) | NR | NR | 6.1 (3.2 - 9.9) | 9.1 (2.8 - 18.1) |
| ***Latin America and the Caribbean (LATAM)*** | | | | | | | | | | | | | |
| *2010-2015* | NR | 61.8 (52.3 - 70.1) | 6.1 (1.7 - 12.5) | 10.8 (4.3 - 19.4) | NR | NR | 0.2 (0.0 - 0.8) | 5.3 (3.2 - 7.8) | NR | NR | NR | 2.8 (0.6 - 6.4) | 13.1 (3.4 - 26.3) |
| *2016-2021* | 0.4 (0.0 - 1.8) | 46.6 (28.3 - 58.3) | 2.5 (0.0 - 7.9) | 35.5 (30.6 - 40.2) | NR | NR | NR | 5.2 (0.0 - 16.4) | 0.5 (0.0 - 1.3) | NR | NR | 1.7 (0.0 - 4.9) | 7.6 (2.9 - 14.0) |
| ***Eastern Europe and Central Asia*** | | | | | | | | | | | | | |
| *2010-2015* | 37.8 (16.3 - 52.3) | 6.8 (3.1 - 11.6) | 0.6 (0.2 - 1.2) | 48.0 (43.2 - 51.4) | 0.2 (0.0 - 0.9) | NR | NR | NR | 1.8 (0.1 - 4.8) | NR | NR | 1.4 (0.2 - 3.2) | 3.4 (0.8 - 7.6) |
| *2016-2021* | 20.9 (14.4 - 28.3) | NR | NR | 68.2 (59.8 - 75.9) | NR | NR | NR | NR | NR | NR | NR | 4.0 (0.5 - 9.9) | 6.9 (1.5 - 15.0) |
| ***Western and Central Europe and North America (WCE/NA)*** | | | | | | | | | | | | | |
| *2010-2015* | 5.8 (2.8 - 9.7) | 50.5 (39.8 - 60.6) | 6.1 (2.3 - 11.4) | 12.7 (1.5 - 31.8) | 1.8 (0.9 - 2.8) | 8.1 (4.6 - 12.5) | 0.5 (0.2 - 0.8) | 3.7 (1.7 - 6.3) | 1.1 (0.5 - 1.9) | 0.2 (0.0 - 1.0) | 0.0 (0.0 - 0.2) | 5.4 (1.7 - 10.9) | 4.1 (1.1 - 8.8) |
| *2016-2021* | 9.8 (0.0 - 36.8) | 64.0 (33.6 - 83.9) | 6.4 (0.0 - 28.8) | 0.5 (0.0 - 2.1) | 3.0 (1.0 - 5.9) | 3.2 (0.0 - 12.7) | NR | 7.2 (3.5 - 12.1) | 0.3 (0.0 - 1.4) | NR | NR | 5.7 (0.0 - 19.8) | NR |

*Other CRFs = any reported CRFs other than CRF01_AE and CRF02_AG; **Other/unknown = subtypes reported by authors as such; NR = Not reported

Supplementary Table 5: Unweighted subtypes reported by country, total sample size (%)

| **Country** | **N, number of papers** | **A** | **B** | **C** | **Other CRFs*** | **CRF, 01_AE** | **CRF, 02_AG** | **D** | **F** | **G** | **H** | **J** | **Other/unknown**** | **URF** |
| --- | --- | --- | --- | --- | --- | --- | --- | --- | --- | --- | --- | --- | --- | --- |
| Algeria | 1 | 0.8 | 12.6 |  | 74.8 |  | 8.4 |  |  |  |  |  | 3.4 |  |
| Angola | 1 | 8.8 |  | 38.2 | 2.9 |  | 5.9 | 5.9 | 17.6 | 8.8 | 2.9 |  | 2.9 |  |
| Argentina | 1 |  | 46.1 |  |  |  |  |  |  |  |  |  |  |  |
| Aruba | 1 |  | 100.0 |  |  |  |  |  |  |  |  |  |  |  |
| Australia | 3 |  | 59.8 |  |  |  |  |  |  |  |  |  | 58.1 |  |
| Austria | 1 | 1.9 | 75.0 | 2.9 |  | 6.7 | 4.8 | 1.9 | 6.7 |  |  |  |  |  |
| Belgium | 2 | 6.8 | 53.2 | 5.8 |  | 3.0 | 16.3 | 1.1 | 10.0 | 15.4 |  |  | 3.1 |  |
| Benin | 2 | 3.3 | 3.8 |  | 7.7 |  | 75.7 |  | 0.4 | 6.5 |  |  | 4.0 | 15.7 |
| Botswana | 1 | 0.4 |  | 99.3 | 0.1 |  |  |  |  |  |  |  | 0.3 |  |
| Brazil | 33 |  | 66.3 | 14.9 | 13.0 |  | 1.1 | 0.4 | 8.6 |  |  |  | 8.8 | 12.9 |
| Burkina Faso | 1 |  |  |  | 33.3 |  | 55.6 |  |  | 11.1 |  |  |  |  |
| Cameroon | 15 | 6.6 | 0.9 | 3.1 | 11.0 | 2.8 | 61.9 | 4.0 | 4.9 | 4.7 | 1.4 |  | 9.1 | 17.1 |
| Canada | 2 |  | 79.4 |  |  |  |  |  |  |  |  |  | 20.6 |  |
| Cape Verde | 1 |  | 4.2 | 1.8 | 4.2 |  | 24.4 |  | 7.7 | 29.2 |  |  |  | 8.3 |
| Chad | 1 | 9.3 |  |  |  |  | 30.2 | 9.3 | 4.7 | 16.3 |  | 30.2 |  |  |
| China | 101 | 1.1 | 11.5 | 4.0 | 38.8 | 41.4 | 0.5 | 0.2 | 0.0 | 0.2 |  |  | 11.6 | 10.6 |
| Cote d'Ivoire | 2 | 9.8 |  |  | 4.9 |  | 85.2 |  |  |  |  |  |  |  |
| Croatia | 1 | 4.2 | 91.6 | 1.7 |  | 0.5 | 1.2 |  |  |  |  |  | 0.7 |  |
| Cuba | 2 | 1.0 | 28.1 | 5.2 | 35.3 |  |  |  |  | 1.4 |  | 1.4 |  | 9.2 |
| Cyprus | 1 | 21.0 | 41.0 | 7.0 | 7.0 |  | 4.0 |  | 8.0 |  |  |  |  | 12.0 |
| Dominican Republic | 1 |  | 98.0 |  |  |  |  |  |  |  |  |  | 2.0 |  |
| DRC | 3 | 19.5 | 1.9 | 19.5 | 9.7 | 1.2 | 5.3 | 2.8 | 3.8 | 7.6 | 10.8 | 1.2 | 31.2 | 23.6 |
| Equatorial Guinea | 1 | 2.6 | 2.6 | 10.5 | 15.8 |  | 55.3 | 2.6 | 2.6 | 2.6 |  |  |  | 5.3 |
| Estonia | 2 | 7.0 | 2.2 | 0.4 | 85.4 |  | 0.4 |  |  |  |  |  | 6.1 | 4.0 |
| Ethiopia | 2 | 1.9 |  | 96.4 |  |  |  |  |  |  |  |  |  |  |
| France | 16 | 4.5 | 50.4 | 3.2 | 4.2 | 2.2 | 24.9 | 1.2 | 2.6 | 1.5 | 0.7 |  | 23.6 | 5.7 |
| Gabon | 3 | 21.1 |  | 1.9 | 12.1 | 3.5 | 20.6 | 6.8 | 0.7 | 14.2 | 5.4 | 0.7 |  | 13.5 |
| Germany | 2 | 6.6 | 72.8 | 4.3 | 1.1 | 2.3 | 4.5 | 0.7 | 1.9 | 2.1 |  |  | 3.4 | 1.1 |
| Ghana | 3 | 2.5 | 5.0 |  |  | 5.6 | 75.6 |  |  | 8.8 |  |  | 12.5 |  |
| Greece | 6 | 39.9 | 32.9 | 2.4 | 11.5 | 1.5 | 5.4 |  |  | 2.1 |  |  | 10.1 | 2.2 |
| Guatemala | 1 |  | 96.6 | 0.7 |  |  |  |  |  |  |  |  |  |  |
| Guinea | 1 | 3.2 |  |  | 9.3 |  | 80.6 | 5.8 |  |  |  |  |  |  |
| Guinea-Bissau | 3 | 3.8 | 1.9 |  | 1.9 |  | 80.8 |  |  | 1.9 |  |  |  |  |
| Honduras | 1 |  | 99.0 | 0.5 |  |  |  |  |  | 0.5 |  |  |  |  |
| Hungary | 2 | 4.4 | 82.2 | 0.6 | 0.6 | 2.6 | 1.5 | 0.6 | 7.3 | 0.6 |  |  | 1.7 |  |
| India | 13 | 2.9 | 5.7 | 91.8 |  | 4.5 |  |  |  |  |  |  | 4.8 | 12.1 |
| Indonesia | 9 | 6.3 | 21.7 |  |  | 62.8 | 2.8 |  |  |  |  |  | 19.2 | 20.3 |
| Iran | 11 | 1.8 | 3.1 | 2.4 | 96.5 | 2.0 |  |  |  |  |  |  | 1.3 |  |
| Israel | 2 | 25.1 | 43.3 | 23.5 |  |  |  |  |  |  |  |  | 4.7 |  |
| Italy | 13 | 2.9 | 64.5 | 3.9 | 6.1 | 1.8 | 8.1 | 0.6 | 6.2 | 2.7 | 0.3 | 0.1 | 25.4 |  |
| Japan | 2 |  | 87.6 | 1.3 |  | 7.3 |  |  |  |  |  |  | 3.9 |  |
| Kazakhstan | 1 |  | 1.3 |  | 1.3 |  | 32.9 |  |  |  |  |  |  |  |
| Kenya | 1 | 65.8 |  | 8.1 |  | 4.7 |  | 16.4 |  | 3.8 |  |  | 9.5 |  |
| Kuwait | 9 | 7.1 | 18.4 | 23.4 | 11.4 | 40.7 | 8.2 |  |  | 4.3 |  |  |  |  |
| Laos | 4 |  |  |  | 8.2 | 91.8 |  |  |  |  |  |  |  |  |
| Liberia | 1 | 1.5 |  | 3.5 | 15.7 | 0.9 | 70.7 | 1.9 |  | 8.8 |  |  |  |  |
| Madagascar | 2 |  |  |  | 100.0 |  |  |  |  |  |  |  |  |  |
| Malawi | 3 | 1.7 |  | 96.6 | 5.3 |  |  | 2.6 |  |  |  |  | 1.6 |  |
| Malaysia | 2 |  | 10.1 |  | 30.9 | 40.9 | 0.7 |  |  | 8.7 |  |  |  | 8.7 |
| Mali | 5 | 10.4 | 2.4 | 3.8 | 11.3 | 8.8 | 69.0 | 5.8 | 1.5 | 4.6 | 1.1 |  | 1.7 | 8.3 |
| Mexico | 3 |  | 96.6 |  |  |  | 2.6 |  |  |  |  |  | 1.2 |  |
| Mongolia | 1 | 2.1 | 47.6 | 2.1 | 14.7 | 2.8 | 8.4 |  |  |  |  |  | 22.4 |  |
| Morocco | 1 |  | 88.5 | 1.3 |  | 1.3 | 9.0 |  |  |  |  |  |  |  |
| Multi-country | 14 | 28.1 | 25.6 | 44.2 |  |  |  | 6.0 | 0.2 | 1.9 |  |  | 5.8 |  |
| Myanmar | 1 |  | 3.4 | 10.3 | 6.9 | 20.7 |  |  |  |  |  |  |  | 10.3 |
| Namibia | 1 |  |  | 96.3 | 0.6 |  | 1.9 |  |  | 1.3 |  |  |  |  |
| Nicaragua | 1 |  | 98.2 | 0.6 |  |  |  |  |  |  |  |  |  |  |
| Niger | 12 |  |  |  | 14.3 |  | 57.1 |  |  | 28.6 |  |  |  |  |
| Nigeria | 1 | 8.1 | 0.5 | 1.4 | 15.7 |  | 48.2 | 0.5 |  | 31.3 |  |  | 18.4 | 2.9 |
| Oman | 3 |  |  |  |  |  |  |  |  |  |  |  | 17.4 |  |
| Pakistan | 3 | 68.6 | 9.8 | 2.9 |  |  | 17.6 |  |  |  |  |  |  | 32.4 |
| Philippines | 1 |  | 16.8 | 1.2 |  | 81.7 |  | 1.9 |  |  |  |  |  |  |
| Poland | 1 | 9.2 | 73.8 | 1.5 |  | 1.0 | 1.0 | 12.9 |  | 0.2 |  |  |  |  |
| Puerto Rico | 3 | 0.1 | 98.9 | 0.1 | 0.4 | 0.2 |  | 0.1 |  |  |  |  | 0.1 |  |
| Romania | 13 | 1.9 | 13.5 | 3.8 |  |  | 0.0 |  | 76.9 | 1.0 |  |  |  |  |
| Russia | 2 | 45.3 | 11.6 | 1.1 | 72.1 | 1.1 | 1.2 |  |  | 4.2 |  |  | 32.0 | 6.0 |
| Rwanda | 1 | 74.0 |  | 26.7 | 1.8 |  |  | 3.3 |  |  |  |  | 22.2 |  |
| Saudi Arabia | 1 | 1.8 | 7.0 | 66.7 |  | 3.5 | 1.8 | 10.5 |  | 8.8 |  |  |  |  |
| Senegal | 1 | 5.6 | 4.6 | 9.3 | 3.7 |  | 71.9 | 2.8 | 3.7 | 1.9 |  |  | 13.5 | 5.6 |
| Serbia | 1 | 1.6 | 93.1 | 2.7 |  | 0.5 | 0.5 |  |  | 0.5 |  |  | 1.1 |  |
| Sierra Leone | 1 | 0.7 | 1.5 | 3.7 | 2.9 |  | 83.1 |  |  | 8.1 |  |  |  |  |
| Slovenia | 15 | 1.6 | 73.8 | 2.1 |  | 0.5 |  |  | 0.5 | 0.5 |  |  |  |  |
| South Africa | 5 | 2.0 | 2.0 | 98.1 |  |  | 0.7 | 0.8 |  |  |  |  | 2.8 |  |
| South Korea | 1 | 0.7 | 89.8 | 0.7 | 1.2 | 4.9 | 1.7 |  |  | 0.9 |  |  | 15.6 |  |
| South Sudan | 7 |  |  | 40.0 |  |  |  | 40.0 |  |  |  |  | 20.0 |  |
| Spain | 1 | 2.7 | 74.5 | 1.0 | 1.0 | 2.9 | 6.0 | 0.8 | 10.4 | 1.7 |  |  | 93.7 |  |
| Sri Lanka | 1 | 7.1 | 12.9 | 51.8 |  | 4.7 | 1.2 | 3.5 |  | 7.1 |  |  | 11.8 |  |
| Suriname | 1 |  | 97.2 |  |  |  |  |  |  |  |  |  | 2.8 |  |
| Sweden | 6 | 8.7 | 26.8 | 21.3 |  | 19.1 | 9.1 |  |  |  |  |  |  |  |
| Taiwan | 3 | 0.9 | 88.3 | 0.9 | 9.6 | 5.2 |  |  |  |  |  |  | 10.3 |  |
| Tanzania | 5 | 40.4 |  | 39.7 | 4.2 |  |  | 7.3 |  |  |  |  | 11.2 |  |
| Thailand | 1 | 9.0 | 10.9 | 5.8 | 6.0 | 80.8 |  |  |  |  |  |  | 13.5 |  |
| Tunisia | 1 | 1.6 | 46.6 | 1.6 | 2.6 |  | 39.4 | 1.0 | 0.5 | 1.0 |  |  |  | 5.7 |
| Turkey | 3 | 7.8 | 49.9 | 4.2 | 36.2 | 1.8 | 13.2 |  | 1.8 | 2.1 |  |  |  |  |
| Uganda | 13 | 43.6 | 1.4 | 4.8 |  | 21.8 | 3.4 | 34.3 |  | 1.0 |  |  | 15.2 | 0.4 |
| UK | 5 | 4.2 | 51.3 | 17.6 | 4.3 | 3.6 | 8.5 | 1.1 | 3.0 | 2.0 |  |  | 8.8 | 11.0 |
| Ukraine | 2 | 93.0 | 5.8 |  | 0.4 |  |  |  |  |  |  |  | 2.1 |  |
| USA | 15 | 0.6 | 94.2 | 1.6 | 0.3 | 1.1 | 1.1 | 0.3 | 0.1 | 0.6 | 0.0 |  | 3.8 | 0.2 |
| Venezuela | 1 |  | 98.1 | 1.9 |  |  |  |  |  |  |  |  |  |  |
| Vietnam | 1 |  | 1.6 |  |  | 97.1 |  |  |  |  |  |  |  |  |
| Zambia | 2 |  |  | 80.6 |  |  | 3.2 |  |  | 3.2 |  |  | 3.2 |  |
| Zimbabwe | 2 | 1.2 |  | 98.2 |  |  |  | 0.6 | 0.6 |  |  |  | 3.0 |  |

*Other CRFs = any reported CRFs other than CRF01_AE and CRF02_AG; **Other/unknown = subtypes reported by authors as such

Bibliography

1. Hemelaar J, Elangovan R, Yun J, Dickson-Tetteh L, Fleminger I, Kirtley S, et al. Global and regional molecular epidemiology of HIV-1, 1990-2015: a systematic review, global survey, and trend analysis. Lancet Infect Dis. 2019;19(2):143-55.

2. Scells H, Zuccon G. searchrefiner: A Query Visualisation and Understanding Tool for Systematic Reviews. Proceeding ed2018. 1939-42 p.

3. Ouzzani M, Hammady H, Fedorowicz Z, Elmagarmid A. Rayyan-a web and mobile app for systematic reviews. Syst Rev. 2016;5(1):210.

4. Wells G, Shea B, O'Connell D, Peterson J, Welch V, Losos M, et al. The Newcastle–Ottawa Scale (NOS) for Assessing the Quality of Non-Randomized Studies in Meta-Analysis. 2000.
